# Early application of high-frequency oscillatory ventilation in H1N1 influenza-related severe ARDS is associated with better outcome

**DOI:** 10.1186/cc10718

**Published:** 2012-03-20

**Authors:** S Jog, M Patel, D Patel

**Affiliations:** 1 Deenanath Mangeshkar Hospital and Research Centre, Pune, India

## Introduction

High-frequency oscillatory ventilation (HFOV) is a promising rescue modality for refractory hypoxia and was used extensively in H1N1 influenza-related ARDS in 2009 and 2010. The aim of this study was to find predictors of successful outcome of HFOV in H1N1 influenza-related severe ARDS [1].

## Methods

Patients with H1N1 influenza-related severe ARDS by the new Berlin definition (applied retrospectively) receiving volume-controlled ventilation (VCV) as per the ARDSnet protocol with PO_2_/FiO_2 _≤100 at PEEP ≥12 cmH_2_O and FiO_2 _≥0.7 were connected to HFOV as a rescue therapy for refractory hypoxia. All patients were followed until discharge from the hospital (survivors) or death (nonsurvivors).

## Results

About 80 parameters were evaluated as outcome predictors of HFOV like demographics, comorbidities, clinical features, laboratory parameters, X-rays, ventilatory and blood gas parameters and therapy-related complications. Previously collected data of 19 patients were analysed applying the new Berlin definition. Demographic, clinical, comorbidity, laboratory and radiological parameters were comparable in survivors and nonsurvivors. Table 1 shows comparison of survivors and nonsurvivors with respect mainly to ventilatory and gas exchange parameters before application of HFOV. Duration of conventional mechanical ventilation before HFOV, 1.4 ± 0.69 versus 3.66 ± 3.53 days (*P *= 0.03), was the only discriminating parameter between survivors and nonsurvivors.

**Table 1:** Comparison of survivors and nonsurvivors

Variable	Survivors	Nonsurvivors	*P *value
APACHE	13.3 ± 1.7	13.2 ± 2.2	0.14
Time VCV	1.4 ± 0.69	3.66 ± 3.5	0.03
PIP	35.6 ± 7.1	35.2 ± 5.1	0.44
PEEP	13.4 ± 2.0	13.4 ± 2.8	0.48
P/F	82.6 ± 31	68.8 ± 34	0.37
OI	36.08 ± 24	25.32 ± 7	0.1

## Conclusion

In H1N1 influenza-related severe ARDS, early application of HFOV is a significant predictor of successful outcome.

## References

[B1] European Society of Intensive Care Medicine Conference Proceedings of 24th Annual Conference of ESICM, Berlin

